# Sterol Characteristics in Silkworm Brain and Various Tissues Characterized by Precise Sterol Profiling Using LC-MS/MS

**DOI:** 10.3390/ijms20194840

**Published:** 2019-09-29

**Authors:** Mika Takeshima, Mari H. Ogihara, Hiroshi Kataoka

**Affiliations:** 1 Department of Integrated Biosciences, Graduate School of Frontier Sciences, The University of Tokyo, 5-1-5 Kashiwanoha, Kashiwa, Chiba 277-8562, Japan; 156333@ibs.k.u-tokyo.ac.jp; 2 National Agriculture and Food Research Organization, 2 Ikenodai, Tsukuba, Ibaraki 305-0901, Japan

**Keywords:** cholesterol, dietary sterol, brain, phytosterol, metamorphosis, LC-MS/MS, principal component analysis, silkworm

## Abstract

Sterols, especially cholesterol (Chl), are fundamental for animal survival. Insects lacking the ability to synthesize Chl are sterol auxotrophic animals and utilize dietary Chl and phytosterols to survive. The sterols obtained from a diet are distributed to the tissues; however, sterol homeostasis in insect tissues remains to be elucidated. This study sought to understand the sterol characteristics of insect tissues through detailed sterol quantification and statistics. The combination of sterol quantification using liquid chromatography tandem mass spectrometry (LC-MS/MS) and principal component analysis (PCA) revealed tissue-specific sterol characteristics in the silkworm, *Bombyx mori*, a phytophagous insect. We found that insect tissues have tissue-intrinsic sterol profiles. The brain has a unique sterol composition as compared to other tissues—high concentration of Chl and less accumulation of phytosterols. Other tissues also have intrinsic sterol characteristics, which when defined by dietary sterols or Chl metabolites, indicate preference for a sterol and consistently manage their own sterol homeostasis. Though most tissues never change sterol profiles during development, the brain drastically changes its sterol profile at the wandering stage, indicating that it could alter sterol composition in preparation for metamorphosis. These results suggest the existence of tissue- and sterol-specific systems for sterol homeostasis in insects.

## 1. Introduction

Sterol is an essential lipid for living organisms. In animals, the main sterol is cholesterol (Chl), which is a necessary component of cell membranes [1], a material of steroid hormones [2], and a ligand for the Hedgehog signaling process [3,4]. Most animals synthesize Chl from acetyl-CoA with sequential enzymatic reactions in all tissues, mainly liver, adrenal gland, brain, ovary, and testis [5]. Chl is predominantly synthesized in the liver, while accumulation of Chl is notable in the brain [6,7]. The breakdown of Chl homeostasis results in severe disease. For example, excessive accumulation of Chl induces atherosclerotic cardiovascular disease and Alzheimer’s disease [8,9,10,11]. Therefore, the maintenance of Chl homeostasis is essential for animal survival.

**Figure 1:**
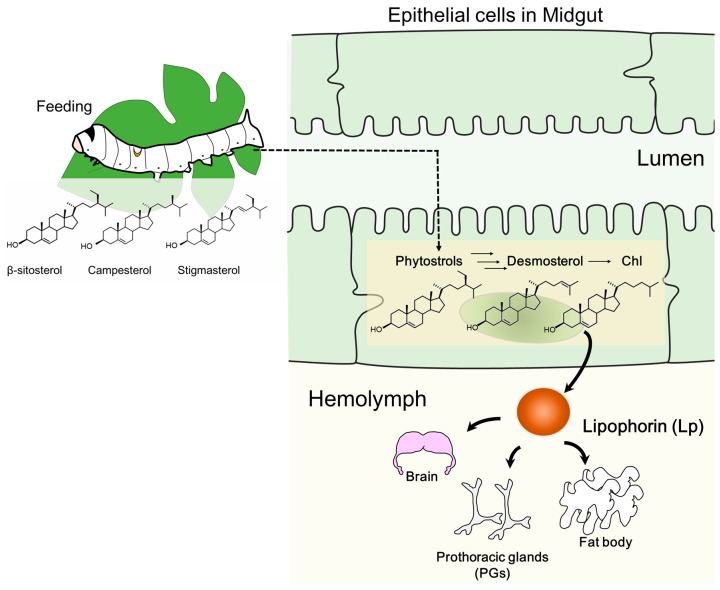
A model of sterol flow in the silkworm. Phytosterols obtained from diet are absorbed into midgut epithelial cells and converted into Chl, which, with other sterols, are transported into insect lipoprotein, lipophorin (Lp). Lp circulation in the hemolymph transports sterols to peripheral tissues.

As in other animals, insects also require Chl for survival; however, they are unable to synthesize Chl de novo in tissues. They lack a series of enzymes that convert squalene to Chl in the Chl synthesis pathway [12,13,14], resulting in a sterol auxotrophic feature. Insects entirely rely on sterol acquisition from their diet—obtaining Chl is, thus, easier for carnivorous insects. In contrast, phytophagous insects need additional mechanisms to obtain Chl, which involves transforming phytosterols, such as β-sitosterol and campesterol, in their diet [15,16]. The silkworm *Bombyx mori*, a phytophagous insect, feeds on mulberry leaves or can be fed an artificial diet. The diet of the silkworm typically lacks Chl, but contains phytosterols [16,17,18]. Once phytosterols from the diet reach the midgut, they are absorbed in the epithelial cells and converted into desmosterol through several enzymatic reaction steps; Chl is finally obtained via the conversion of desmosterol by 3β-hydroxysteroid-Δ24 reductase (Figure 1) [19,20]. From the midgut cell to the hemolymph, Chl is transferred to a major insect lipoprotein, lipophorin (Lp), which has an intermediate feature of mammalian high- and low-density lipoproteins [21,22,23,24,25]. In addition to Chl, Lp transports other lipids, fatty acids, diglyceride, and a small amount of phytosterols [22,23,26]. As major dietary sterols, phytosterols (β-sitosterol, campesterol, stigmasterol) are detected from Lp in the silkworm [26]. Insect tissues incorporate sterols from Lp and maintain sterol homeostasis (Figure 1), the disruption of which causes severe damage [27,28,29,30]. Brain and prothoracic glands (PGs, a pair of organs for insect steroid hormone synthesis) are known to be Chl-rich tissues [17]. Disruption of sterol homeostasis in these tissues causes their malfunctioning, and subsequent fatality [28,29,30,31]. Null mutant or RNAi of Niemann-Pick disease type C1 (dNPC1) of *Drosophila melanogaster* showed abnormal accumulation of Chl in the brain, causing neurodegenerative symptoms and shortening of its lifespan [28]. In addition, conditional knockdown of the factors required for Chl homeostasis in the organ for steroid hormone synthesis causes disruption [29,31]. Ecdysteroid, a solo steroid hormone in insects, is required for molting and metamorphosis, and is synthesized in PGs, or a ring gland for *Drosophila*. Generally, ecdysteroid is synthesized from Chl through multistep conversion. Chl is converted into 7-dehydrocholesterol (7dC) at the first enzymatic reaction, and then finally transformed into ecdysone, a prohormone of ecdysteroid in the gland. This prohormone is secreted into the hemolymph and converted into the active form, 20-hydroxyecdysone (20E). Disruption of Chl homeostasis in the ring gland of *Drosophila* causes loss of ecdysteroid synthesis and developmental arrest. As shown in these reports, the maintenance of Chl homeostasis in insect tissues is essential for survival.

Insect tissues obtain sterols from the Lp through receptor-mediated endocytosis [32,33,34]. Lp receptor (LpR) is ubiquitously expressed in insect tissues [35]; therefore, LpR-mediated endocytosis is considered an essential mechanism for Chl incorporation into insect tissues. However, the process of Chl incorporation from the Lp appears to be different for each tissue. Igarashi et al. [36] visualized Chl incorporation into silkworm tissues using different fluorescently labelled Lp and Chl, respectively. In the brain, Lp and Chl signals were co-localized in the cytoplasmic region, while in the PGs, Chl signals in the cytoplasmic region were separated from Lp signals in the cell membrane [36]. Therefore, insect tissues may have tissue-specific mechanisms for Chl incorporation from Lp and maintenance of Chl homeostasis. Interestingly, insects can accumulate other dietary sterols in their tissues [17,18,31,37,38]. Phytosterols in dietary sterols are mainly utilized for Chl synthesis in the midgut, but accumulation of these sterols in other tissues has also been reported for *Drosophila* [31]. The accumulation of dietary sterols in silkworm tissues (as well as in *Drosophila*), especially adipose tissue (named the fat body), has been reported [17,18]. These reports highlight the fact that insect tissues can accumulate phytosterols as well as Chl. Recently, Lavrynenko et al. [37] revealed that modification of sterol proportions in an artificial diet affect the types of ecdysteroids synthesized in *Drosophila* using LC-MS/MS. Unlike other insects, *Drosophila* can synthesize several types of ecdysteroids, such as 20E and Makisteron A, which is methylated 20E at the C-24 position. When they are reared on an artificial diet with different sterol composition, the synthesized ecdysteroids are different; a diet with Chl induces synthesis of 20E, the common form of ecdysteroids in insects, while a diet with phytosterol, with methylation at the C-24 position, induces synthesis of Makisteron A. These results indicate that dietary sterols are utilized in the ring gland of *Drosophila* as materials for ecdysteroid synthesis. Other tissues or other insects, like the *Drosophila* ring gland, may utilize dietary sterols directly or convert them to another steroid without converting Chl.

Several studies have been conducted to elucidate the universal mechanism of Chl incorporation and maintenance in insect cells [39,40,41,42]; however, sterol homeostasis in insect tissues could be maintained by the integration of sterol-specific management systems in the respective tissues. Fewer studies have focused on tissue-specific and sterol-specific mechanisms. The lack of information about sterol profiles in insect tissues prohibits a proper understanding of sterol homeostasis. To clarify the sterol profiles of insect tissues, we developed methods to detect and quantify multiple sterols by multiple reaction monitoring (MRM) using LC-MS/MS [17,43]. Three major phytosterols (β-sitosterol, campesterol, and stigmasterol), ergosterol, desmosterol (a precursor of Chl), and Chl were detected from insect tissues simultaneously using this method [17]. To understand sterol homeostasis in insects, further consideration of Chl metabolites are necessary. Chl is rarely metabolized in the insect body, but is converted to ecdysteroids [44]. Ecdysteroid synthesis is a multistep reaction and most of its intermediates have been clarified. Among the intermediates of ecdysteroid, only 7dC was detected in the PGs [43]. In this study, we quantified Chl and six other sterols in various tissues of the silkworm—from the final-instar larvae until pupation—and characterized developmental sterol profiles in the respective tissues. Our results demonstrate that the silkworm brain is able to change its sterol profile at the beginning of the post feeding period, just ahead of the morphological change to pupae. This is the first report highlighting the fact that an insect’s brain has a critical time point to alter sterol characteristics.

## 2. Results

### 2.1. Changes in Sterol Composition in the Midgut and Hemolymph as the Suppliers of Sterols to Other Tissues

In this study, we determined the sterol profiles of silkworm tissues and hemolymph. The brain and PGs tissues were very small, dissected from individual silkworms, and used for sterol extraction. We also dissected and utilized the other larger tissues. We homogenized the samples in a buffer and extracted sterols from the supernatant of the homogenate by the Bligh and Dyer extraction method. Direct extraction of sterols by the Bligh and Dyer method was not done because the tissues were small and the amount of sterol in each sample required to be normalized by the amount of protein, instead of wet weight or dry weight. There was no significant difference in extraction efficiency between our method and the direct Bligh and Dyer method (Figure S1). We set LC conditions and the parameters of MRM for LC-MS/MS to detect and quantify nine sterols, as mentioned in Materials and Methods. Four dietary sterols, including three phytosterols (β-sitosterol, campesterol, and stigmasterol) and ergosterol, desmosterol (a precursor of Chl), Chl, and metabolites of Chl (7dC) were measured from tissues of the silkworm. For hemolymph, methanol extraction (which is commonly used for ecdysteroid extraction), was used (Figure S2). We measured dietary sterols, desmosterol, Chl, and metabolites of Chl, including ecdysone and 20E, in the silkworm hemolymph. Since ecdysone and 20E were not contained in any silkworm tissues, we quantified ecdysone and 20E only in the hemolymph.

We used final-instar larvae to investigate the developmental sterol profiles of the silkworm. The silkworm never feeds after the mid final-instar phase, even in an adult stage; therefore, the first half of the final instar phase is an important period to obtain nutrients for the rest of its life. The silkworm strain used in this study stopped feeding at day 6 and initiated a wandering behavior in preparation of pupation. We defined the former period before wandering and spinning as the “feeding period” and the subsequent fifth instar period as the “post-feeding period”.

**Figure 2:**
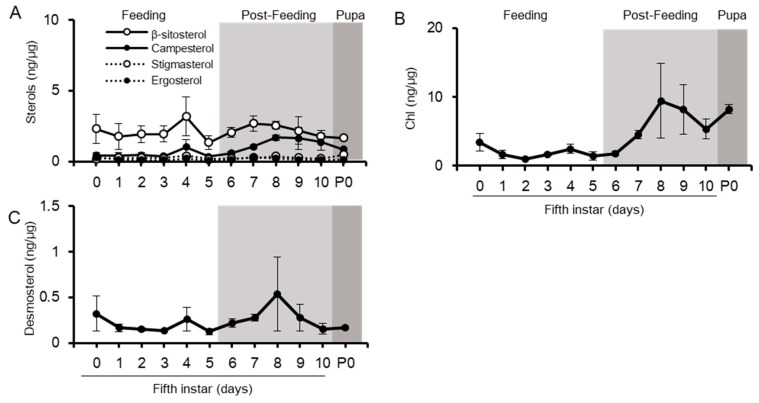
Changes in sterol amounts in the midgut from final instar to pupal periods. The amount of dietary sterols (**A**), Chl (**B**), and desmosterol (**C**) was quantified using LC-MS/MS and standardized using the protein amounts of the respective samples. Values are mean ± s.d. (*n* = 3). P0: pupal stage day 0.

**Figure 3:**
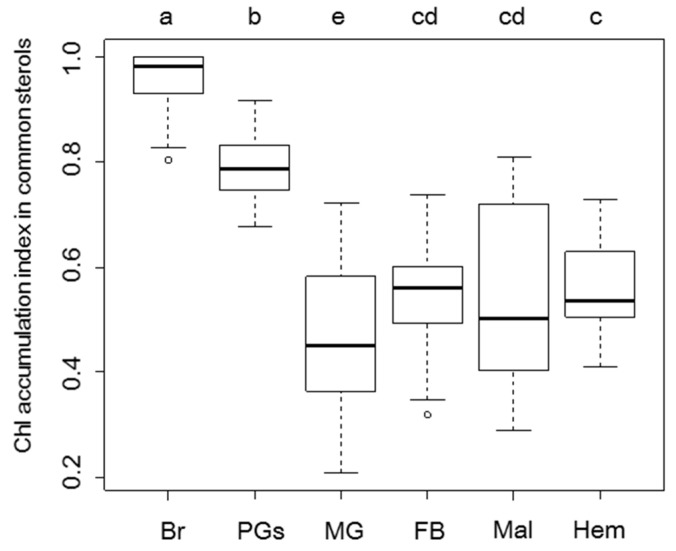
Comparison of Chl proportion to the amount of common sterols in silkworm tissues and the hemolymph. Dietary sterols (β-sitosterol, campesterol, stigmasterol, and ergosterol), desmosterol, and Chl were detected in most tissues and hemolymph. Chl accumulation index in common sterols showed the Chl proportion compared to common sterols. The data in this figure are the same set as in Figure 2, Figure 4, Figure 5 and Figures S6 and S7. The statistical significance of differences was determined by one-way analysis of variance (ANOVA) followed by Tukey-Kramer’s test. Br: brain, PGs: prothoracic gland, MG: midgut, FB: fat body, Mal: Malpighian tubules, Hem: hemolymph.

The midgut of the silkworm is the main tissue for conversion of phytosterols to Chl and the initial resource of dietary sterols to other tissues (Figure 1). As expected, dietary sterols (β-sitosterol, campesterol, stigmasterol, and ergosterol), desmosterols, Chl, and small amount of Chl metabolites (7dC) were detected in the midgut (Figure 2 and Figure S3C). Chl was the dominant sterol in the midgut and accumulated much more than the phytosterols (Figure 2A,B). The Chl accumulation index of common sterols showed the proportion of Chl to accumulated sterols in each tissue (Figure 3 and Figure S4); Chl proportion in the midgut accounted for almost half of the common sterol amount. In the post-feeding period, β-sitosterol and campesterol amounts gradually decreased, while the amount of Chl increased in this period, indicating that the midgut continued to convert phytosterols into Chl, even in the post-feeding period.

**Figure 4:**
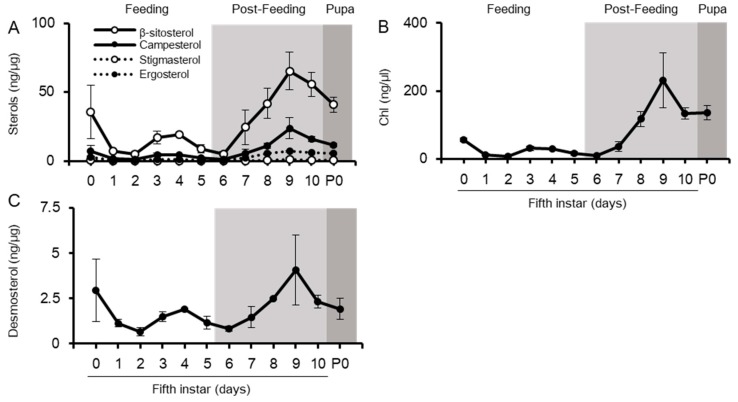
Changes in sterol concentrations in the hemolymph from final instar to pupal periods. The concentrations of dietary sterols (**A**), Chl (**B**), and desmosterol (**C**) were quantified. Values are mean ± s.d. (*n* = 3). P0: pupal stage day 0.

It is possible that sterols in the midgut, including dietary sterols and Chl, might be transferred to the lipophorin (Lp) in the hemolymph (Figure 1). The silkworm hemolymph contains dietary sterols, desmosterol, and Chl, which were detected in the midgut (Figure 2 and Figure 4). In addition, the hemolymph contains Chl metabolites, 7dC, ecdysone, and 20E, which might be secreted from the PGs (Figures S3B and S5). Though hemolymph contains dietary sterols and Chl metabolites, the major sterol is Chl (Figure 4). It appears to be actively transported to the hemolymph, probably to Lp, while other sterols are incidentally transported to the hemolymph. Unlike the midgut, all detected sterols in the hemolymph increase their concentration in similar form during the post-feeding period, indicating the high activity of transportation of sterols with the same ratio from the midgut to the hemolymph, even in the post-feeding period.

### 2.2. Sterol Profiles in Various Tissues

**Figure 5:**
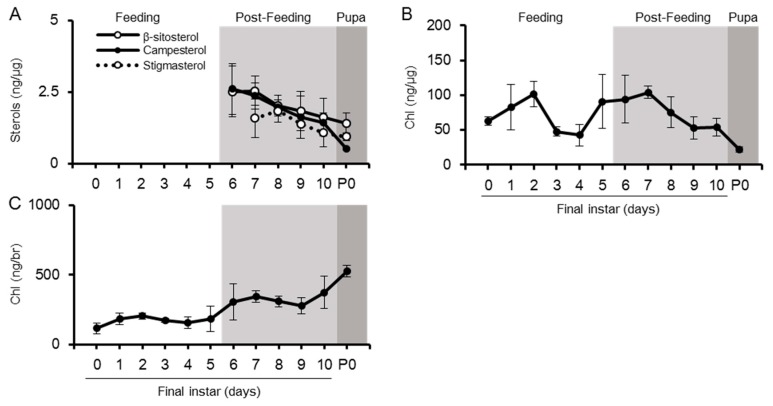
Changes in sterol amount in the brain from final instar to pupal periods. The amount of dietary sterols normalized by protein amounts (**A**), the amount of Chl normalized by protein amounts (**B**), and Chl amounts in a single brain (**C**). Values are mean ± s.d. (*n* = 3). P0: pupal stage day 0.

We quantified sterol amounts in the brain and other recipient tissues from Lp. In the brain, Chl is the predominant sterol, with a lack of desmosterols and Chl metabolites (Figure 5). Dietary sterols were not detected during the feeding period, but β-sitosterol, campesterol, and stigmasterol appeared once in the post-feeding period (Figure 5A). They were, however, lower in quantity than Chl (Figure 5A,B), which had the highest Chl accumulation index (Figure 3). Therefore, the brain seems to have a potent preference for Chl: the amount of Chl in a single brain showed gradual increase, particularly on transition from the feeding period to the post-feeding period. This amount is five times larger in the pupal stage than in the first day of the fifth instar. (Figure 5C). However, when sterols were normalized with protein, a clear increase in Chl amount was not observed (Figure 5B). The uptake of Chl by the brain may cause its enlargement during the final instar development, thus maintaining a certain ratio of range to fit brain sizes. In contrast to Chl, clear change in the amounts of dietary sterols in the brain was detected after the post-feeding period (Figure 5A). The brain clearly changed its sterol profile during the development cycle (Figure 5).

**Figure 6:**
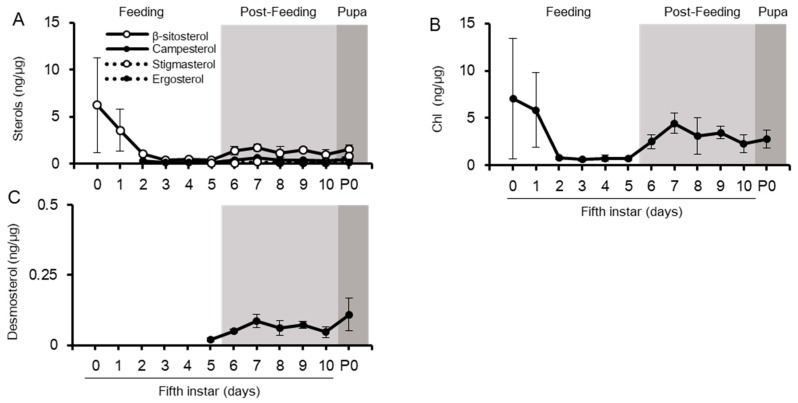
Changes in sterol amount in the fat body from the final instar stage to pupal periods. The amount of dietary sterols (**A**), Chl (**B**), and desmosterol (**C**). Each sterol amount was normalized using the protein amounts of respective samples. Values are mean ± s.d. (*n* = 3). P0: pupal stage day 0.

To compare sterol profiles, quantification of sterols in the fat body was conducted (Figure 6). The fat body is insect adipose tissue; it has an irregular shape and exists in various places in the insect body. In the fat body as well as in the brain, Chl was the major sterol observed; however, phytosterols were detected in all stages (Figure 6). The fat body contained desmosterols from the last half of the fifth instar. Although the fat body had phytosterols even at the feeding period, their profiles were different from those of the brain: the fat body had β-sitosterol as the major phytosterol, followed by other phytosterols, while the brain had similar amounts of the three phytosterols. The ratio of phytosterols in the fat body was similar to that in the midgut and hemolymph, rather than that in the brain. Desmosterol, an intermediate for Chl synthesis, was also detected in the fat body. Chl accumulation index in common sterols was much lower in the fat body than that in the brain (Figure 3). We also quantified sterols in other major tissues—prothoracic glands (PGs) and Malpighian tubules (the equivalent of kidneys in insects) (Figures S6 and S7). These tissues contained both Chl and phytosterols. Though these tissues contained large amounts of Chl, its accumulation index in common sterols in the PGs and Malpighian tubules were lower than that of the brain (Figure 3). These results indicate that most silkworm tissues incorporate and maintain Chl and phytosterols, but the brain selectively accumulates Chl during the feeding period. In addition to Chl and phytosterols, the PGs contained 7dC, a metabolite of Chl in the process of ecdysteroid synthesis. In the PGs, Chl was the major sterol among common sterols (dietary sterols, desmosterols, and Chl) in PGs, as shown in Figure 3. In contrast, the proportion of Chl in total sterols was the lowest in the PGs, compared to other tissues (Figure S4), due to the large accumulation of 7dC in the PGs. Since 7dC is synthesized from Chl with a single enzymatic reaction, these results suggest that the PGs require large amounts of Chl, similar to the brain. In addition to Chl and dietary sterols, the PGs might maintain 7dC accumulation for their functions.

We also compared the developmental changes of sterol profiles among tissues. The amounts of Chl and β-sitosterol in the fat body decreased through the feeding period and increased during the post-feeding period. Other sterols, campesterol, stigmasterol, desmosterol, and ergosterol, were low but detectable in the feeding period and increased during the post-feeding period. These patterns of phytosterols were similar to those of the hemolymph during the post-feeding period, but were not observed in the brain. The patterns of phytosterols in PGs and Malpighian tubules did not show any obvious change in dietary phytosterols during feeding and post-feeding periods. Results of sterol profiles and developmental patterns in the fat body, the PGs, and the Malpighian tubules emphasize this specific feature of the brain in both feeding and post-feeding periods.

### 2.3. Visualization of Tissue Characteristics of Sterol Profiles Using Principal Component Analysis

**Figure 7:**
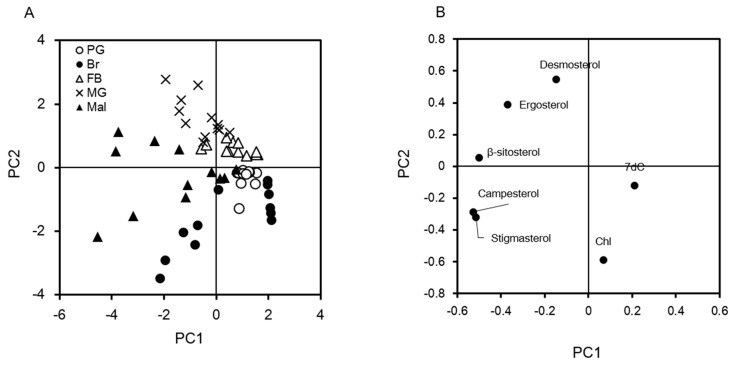
Characterization of insect tissues by steroid profiles using principal component analysis (PCA). The averages of dietary sterols, desmosterol, Chl, and 7dC in respective tissues (Figure 2, Figure 5, Figure 6, and Figures S3A,C, S6 and S7 ) were analyzed by PCA, and are shown in a score plot (**A**) and loading plot (**B**). Score plot represents the characterization of tissues in respective stages determined by sterol profiles. Loading plot represents the characteristics of sterols extracted from the data. The vertical axis is the first principal component and the horizontal axis is the second principal component.

As seen above, the brain and other tissues had different sterol profiles (four dietary sterols, desmosterols, Chl, and 7dC). We analyzed these profiles by chemometrics using principal component analysis (PCA), a form of multivariate analysis, to visualize the characteristics of each tissue. In addition to sterol profile of the brain (Figure 5), those of the midgut (Figure 2 and Figure S3C), the fat body (Figure 6), the PGs (Figures S3A and S6), and Malpighian tubules (Figure S7), were also utilized for PCA (Figure 7). In PCA analysis, respective tissues were plotted at nearby sites but did not overlap with other tissues (Figure 7A). The midgut and the fat body were plotted relatively close, while other tissues, such as the PGs, were plotted apart from the midgut and the fat body, meaning insects have tissue-intrinsic sterol characteristics.

A loading plot (Figure 7B) demonstrated distinctive sterols, which determine the characteristics of tissues in Figure 7A. We found that the brain was characterized by Chl amounts. In contrast, the midgut and the fat body were characterized by the amount of ergosterol and desmosterol, respectively. Malpighian tubules were characterized by the presence of campesterol and stigmasterol. In terms of the characteristics of PGs, their primary features were the appearance of 7dC and their high level of Chl. The sterols that define the characteristics of the respective tissues may the key to understand sterol homeostasis in them. Interestingly, the brain was separated into two groups, feeding and post-feeding periods, while no other tissues showed such developmental differences (Figure S8A). The change of sterol characteristics in the brain was due to the appearance of campesterol and stigmasterol during the post-feeding period. These results indicate that the brain could change sterol incorporation of these sterols before pupation.

## 3. Discussion

In this study, we quantified seven sterols in silkworm tissues by MRM using LC-MS/MS, and characterized each tissue based on sterol profiles. The PCA of sterol profiles in various tissues revealed that each tissue has similar sterol characteristics through the fifth-instar larvae stage, but only the brain clearly changed sterol characteristics between the feeding and post-feeding periods during the fifth-instar larvae period.

Silkworm brain showed higher Chl accumulation than other tissues in both feeding and post-feeding periods (Figure 5 and Figure 7) and was basically a highly Chl auxotrophic tissue, even though insect brain does not have myelin sheath, a Chl-rich region in the brain of mammals. Although the amount of Chl in a single brain tissue gradually increased, the amount of relative Chl (normalized with protein content) showed fluctuation in a specific range. The increase in Chl content could correspond to enlargement of the brain. Chl accumulation in the brain might have occurred in the cell membrane of neurons, because elongation and remodeling of nerve axons take place during larval development and metamorphosis in insects [45]. Excessive accumulation of Chl induces malfunctioning of the brain; neurodegenerative diseases are a result of this [28]. A silkworm brain is expected to increase Chl amounts to maintain Chl homeostasis from the fifth instar to pupal stages.

Chl is the predominant sterol in the brain, but the transition of sterol characteristics is due to the increase of phytosterols (β-sitosterol, campesterol, and stigmasterol) in the beginning of post-feeding of the fifth instar larvae. This change was not associated with the sterol characteristics of the hemolymph containing Lp as the exogenous sterol source (Figure 4 and Figure 5). Our previous paper demonstrated that Lp and Chl were co-localized in the cytoplasm in the brain on day 4 in the fifth instar stage [36]. Lp was able to transport both Chl and phytosterols [26]; thus, the brain mainly obtains sterols through the endocytosis of Lp, which occurs in a less-selective manner [36]. Therefore, high Chl and low phytosterol accumulation in the brain may indicate active endocytosis of Lp and high selective excretion of phytosterols. In mammals, Hedgehog signaling is involved in the regulation of Chl efflux and its activation inhibits the excretion of Chl from mammalian cells [46]. In *Drosophila*, Hedgehog signaling is activated in the late larval stage and acts in the larval brain [47]. Hedgehog signaling might be related to the excretion of phytosterols in the brain. A comparison of RNA-seq data of the brain on the first day of the post-feeding period with that of the feeding period should help identify the essential factors and mechanisms involved in inducing change in sterol characteristics. Clarification of the distribution of Chl and phytosterols in the brain will also help understand their incorporation and usage.

The transition of sterol characteristics in the brain takes place in the first day of the post-feeding period; therefore, the brain gets a cue to change sterol composition prior to the morphological change to pupae. One such signal comes from 20E, an active form of ecdysteroid, because it regulates morphological changes such as neuronal apoptosis and remodeling of nerve axons in the brain [48,49]. Although the change in sterol characteristics in the brain was not synchronized with the peak of 20E titer in the hemolymph (Figure S5), a wandering behavior at the beginning of the post-feeding period is thought to be induced by a small increase of the 20E titer [50,51,52,53]. Therefore, the change in sterol characteristics in the brain might be induced by 20E. To clarify this influence, further study on the factors required for sterol incorporation and maintenance is required.

Like the brain, other tissues also have intrinsic sterol characteristics. While the amount of each sterol in various tissues varies through development stages, the PCA revealed that the sterol characteristics of each tissue were similar during the fifth instar larvae stage (Figure 7), but had specific differences. The sterol characteristics of the fat body were shown to resemble those of the midgut, indicating that sterols accumulated less selectively from the midgut through the hemolymph (Figure 2, Figure 4, Figure 6 and Figure 7). The Chl accumulation index in common sterols was similar among Malpighian tubules, the fat body, and the hemolymph (Figure 3); however, the PCA results showed that Malpighian tubules had different sterol characteristics due to the presence of stigmasterol and campesterol (Figure 7). Malpighian tubules, which correspond to the kidney in mammals, contains a significant amount of phytosterols. Because the feces of silkworm contain phytosterols, especially stigmasterol (unpublished data), Malpighian tubules selectively accumulate and excrete these phytosterols. As for the PGs, the tissues showed a high preference for Chl and 7dC (Figure 7, Figures S3A and S6). This should be linked to their function as an ecdysteroidogenic tissue, and the PGs might possess a management system that allows high retention of Chl and 7dC [54]. Interestingly, 7dC was detected from the hemolymph and its concentration was higher than that of ecdysteroids (Figures S3B and S5). Although 7dC is recognized as an intermediate of ecdysteroids in insects, it is also known as a precursor of Vitamin D in animals; thus, 7dC circulating in the hemolymph might have other important functions as well.

Several studies have shown that Lp is incorporated through LpR-mediated endocytosis [33,34]. However, the intracellular behaviors of Lp and Chl differ in silkworm tissues [36]. Recent studies have shown that two members of the scavenger receptor class B type 1 (SR-B1) are required for the selective uptake of carotenoids derived from Lp in silk glands [55,56,57]. These SR-B1s showed gene expression in limited tissues, while LpR is expressed ubiquitously [35]. Similar to carotenoids, specific receptors for dietary sterols, and probably Chl, might be present in insect tissues for sterol-specific incorporation. In insects, there is little study on the excretion of sterols from peripheral tissues. The ABC transporters—ABCG5 and ABCG8—are known to be required for excretion of phytosterols in mammals [58]. Insects possess orthologues of these ABC transporters [59,60]; however, their roles have not been clarified yet. Functional analyses of the transporters and other factors will be required for a comprehensive understanding of sterol homeostasis in insect tissues.

In conclusion, our results clearly show that the silkworm brain can change sterol characteristics at specific times in their development cycle. In addition, silkworm tissues maintain sterol homeostasis in a tissue-specific manner. There isn’t enough information available on this tissue-intrinsic sterol homeostasis, which could be common in insects and other animals. The tissues (mid-intestine, liver, and muscle) of Atlantic salmon have exhibited different compositions and amounts of phytosterol [61]. Tissue-specific management, incorporation, and excretion of dietary sterols might be universal across the animal kingdom. Insects are required to maintain sterol homeostasis by simple systems, sterol incorporation, and excretion because they lack the ability to synthesize sterols. Insect tissues are a powerful model for the study of the mechanisms to incorporate and excrete Chl and phytosterols. The analysis used in this study was successful in characterizing tissues by sterol profiles. This information will help identify new factors required for the maintenance of sterol homeostasis in the post-genomic era.

## 4. Materials and Methods

### 4.1. Chemicals

β-sitosterol (>98% purity), campesterol (>65% purity), desmosterol (>85% purity), ergosterol (>95% purity), cholesterol (>99% purity), 7-dehydrocholesterol (>99% purity), ecdysone (>90% purity), and 20-hydroxyecdysone (>93% purity) were purchased from Sigma-Aldrich (St. Louis, MO, USA). Cholesterol-3,4-_13_C_2_ (>99% purity) was purchased from C/D/N Isotopes, Inc (Pointe-Claire, Quebec, Canada). Stigmasterol (>99% purity) was purchased from Tama Biochemical Co (Tokyo, Japan).

### 4.2. Silkworm

Larvae and pupae of silkworm, *Bombyx mori* (racial hybrid Kinshu×Showa), were used in this study. Silkworm larvae were reared on an artificial diet, SilkMate PS (Nihon Nosan Kogyo, Yokohama, Japan), throughout their life cycle. Larvae and pupae were reared and kept at 25 °C under a 16-h light/8-h dark photoperiod. After hatching of the eggs, silkworm larvae take about 1 month for adult emergence through six molts. To minimize variation in nutrient conditions, larvae just before molting were starved until all larvae for examination were molted. On the final day for staging, fourth-instar larvae were not given access to a diet. Newly molted final-instar larvae were starved until all larvae for examination had molted. In this study, newly molted larvae before feeding were used as day 0 of the fifth-instar larvae, to set the same nutritional conditions. Final-instar larvae were fed until day 5. As preparation for pupation, feeding was terminated and the larvae exhibited wandering behavior on day 6. Larvae pupated on day 11.

### 4.3. Extraction of Sterols from Silkworm Tissues and Hemolymph

The brain, a pair of PGs (ecdysteroidogenic organ), the midgut, the fat body (adipose tissue), and Malpighian tubules (elimination tissue) were dissected from the final-instar larvae to pupae stages. The samples were stored individually at −80 °C until sterol extraction. Isolated tissue was homogenized with close-fitting plastic pestles and sonicated for 10 min in a buffer containing 50 mM Tris-HCl, pH 7.5, 150 mM NaCl, and 2 mM EGTA. Homogenized samples were centrifuged at 5000 rpm for 10 min at 4 °C. The obtained supernatant was used for steroid extraction and protein quantification (normalization of tissue sizes). A part of the supernatant was used for protein quantification with the Bradford method using a protein assay (Bio-Rad Laboratories, Inc. Hercules, CA, USA). For the sterol extraction, 100 ng of cholesterol-3,4-_13_C_2_ in methanol was added to the rest of the supernatant. Sterols, including the internal standard, were extracted using chloroform/methanol (chloroform/methanol/sample: 10/10/9) by the Bligh and Dyer method [62]. The organic layer was collected and evaporated to dryness in a centrifugal evaporator. The samples were re-suspended with 500 μL of methanol or 120 μL for the brain and the PGs.

To measure the sterol concentrations of silkworm hemolymph, hemolymph was collected from day 0 to day 10 of fifth-instar larvae and pupae. Sterols in the hemolymph were extracted with nine volumes of 100% methanol. The samples were shaken vigorously and centrifuged at 3000× *g* for 10 min. The supernatant was evaporated to dryness and re-suspended with 250 μL of methanol.

### 4.4. Quantitation of Sterols in Silkworm Tissue or Hemolymph Using LC-MS/MS

Sterol quantification was performed by MRM using LC-MS/MS with the same conditions as reported previously [17,43], with minor modifications. Briefly, Chl, β-sitosterol, campesterol, stigmasterol, and ergosterol, desmosterol and 7dC in the tissues and the hemolymph were separated with a Pegasil ODS column (3 µm, 2.0 mm × 100 mm; Senshu Scientific Co., Ltd., Tokyo, Japan.) at a flow rate of 0.3 mL/min using isocratic flow of acetonitrile. Ecdysone and 20E in the hemolymph were separated with a Pegasil ODS column (3 µm, 2.0 mm × 50 mm; Senshu Scientific Co., Ltd., Tokyo, Japan) at a flow rate of 0.4 mL/min using acetonitrile and water with gradient elution. For quantification of the targeted steroids, 10 µl of standards and samples were injected. A mixture of standard chemicals was used for the creation of standard curves at concentrations from 0.8 to 1000 ng/mL. All stock solutions were stored at −20 °C until use. Separation and detection of sterols were performed using a Prominence Gradient HPLC system (Shimadzu, Kyoto, Japan), combined with a triple quadrupole QTRAP 5500 mass spectrometer (AB SCIEX, MA, USA).

### 4.5. Statistical Analysis

Statistical analysis for sterol data was performed with R (version x64 3.4.3). PCA was performed with the prcomp package for the raw data of all targeted sterols in five tissues. In the PCA analysis, values under the detection limit in the data were replaced with zero. Significant differences in the Chl proportion in each tissue were analyzed by one-way analysis of variance (ANOVA), followed by Tukey-Kramer’s test.

## 5. Conclusions

This study quantified sterols in insect tissues using LC-MS/MS and following PCA analysis successfully clarified sterol characteristics in insect tissues. Insect tissues contained dietary sterols in addition to large amounts of Chl. Our results showed minor sterols could determine sterol characteristics in tissues, like stigmasterol in Malpighian tubules. Further studies for sterol homeostasis in insect tissues need to consider the maintenance of minor sterols as well as Chl. Silkworm tissues kept their intrinsic sterol characteristics through final instar to pupal periods. In contrast, the brain changed the sterol characteristics drastically at specific times, just before wandering, by the sudden appearance of phytosterols. There might be differences of regulatory systems of sterol homeostasis in the brain at the timing. Further analysis to find related factors for this change in the brain will help to understand sterol homeostasis and some disease caused by abnormality in sterol homeostasis.

## Supplementary Materials

Supplementary materials can be found at https://www.mdpi.com/1422-0067/20/19/4840/s1. Figure S1. Comparison of two methods for sterol extraction from the silkworm brain. Figure S2. Comparison of two methods for sterol extraction from the hemolymph of the silkworm. Figure S3. Change of the 7-dehydrocholesterol (7dC) amounts in silkworm tissues and the hemolymph. Figure S4. Comparison of Chl proportion to total sterols in silkworm tissues and the hemolymph. Figure S5. Changes of ecdysone and 20-hydroxyecdysone (20E) concentrations in the hemolymph. Figure S6. Changes of sterol amounts in the PGs from final instar to pupal periods. Figure S7. Changes of sterol amounts in Malpighian tubules from final instar to pupal periods. Figure S8. Characterization of insect tissues by sterol profiles with labels of developmental stages.

## Abbreviations

Chl	Cholesterol
LC-MS/MS	liquid chromatography tandem mass spectrometry
PCA	principal component analysis
PGs	prothoracic glands
MRM	multiple reaction monitoring
Lp	Lipophorin
7dC	7-dehydrocholesterol
20E	20-hydroxyecdysone
SCRB	scavenger receptor class B type 1
LpR	lipophorin receptor
