# Supplementary material for: Sterol Characteristics in Silkworm Brain and Various Tissues Characterized by Precise Sterol Profiling Using LC-MS/MS
Source: Int J Mol Sci. 2019 Sep 29;20(19):4840. doi: 10.3390/ijms20194840 (PMC6801466; doi:10.3390/ijms20194840)
Supplement: Supplementary file 1 [file ijms-20-04840-s001.pdf]

Supplementary Figures

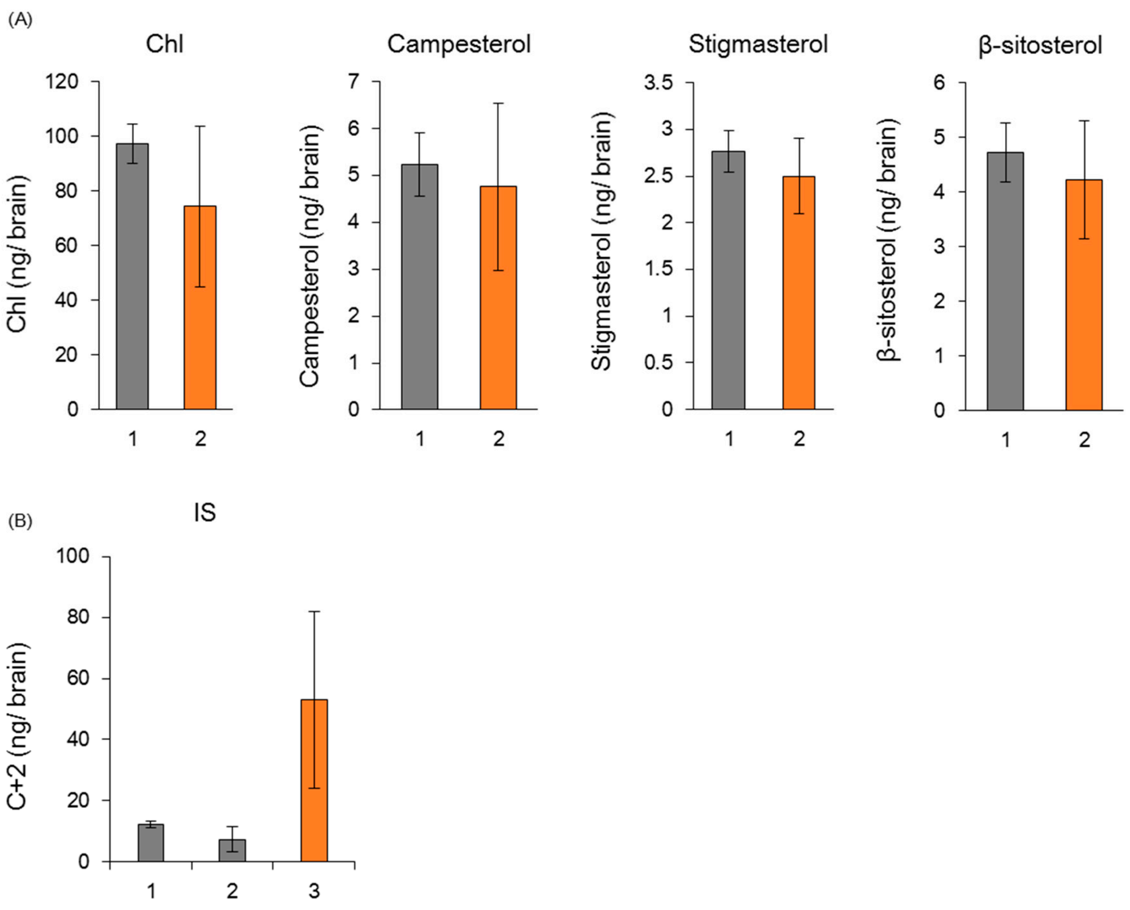

Figure S1. Comparison of two methods for sterol extraction from the silkworm brain. (**A**) The amount of sterols extracted from the silkworm brain. Values are mean ± s. d. (n = 3). The sterols were extracted from the brain of the fifth instar larvae (Day 7) as follows. [1] methanol/chloroform (1:1, v/v) was added to the brain samples before homogenization; [2] the brain was homogenized in the buffer, containing 50 mM Tris-HCl, pH 7.5, 150 mM NaCl, and 2 mM EGTA, and then chloroform/methanol were added to buffer (chloroform/methanol/buffer: 10/10/9, v/v) for sterol extraction. The analysis of steroids were performed using LC-MS/MS as described in Materials and Methods section. Desmosterol, ergosterol, 7dC, ecdysone, and 20E were not detected from any samples. (**B**) The amount of internal standard (IS), which was extracted as follows. [1] IS was added to the brain sample, then extracted using methanol/chloroform, which was added directly to the samples; [2] IS was added to the brain sample before homogenization in the buffer, and extracted using methanol/chloroform; [3] IS was added to the brain samples after homogenization in the buffer, and extracted using methanol/chloroform.

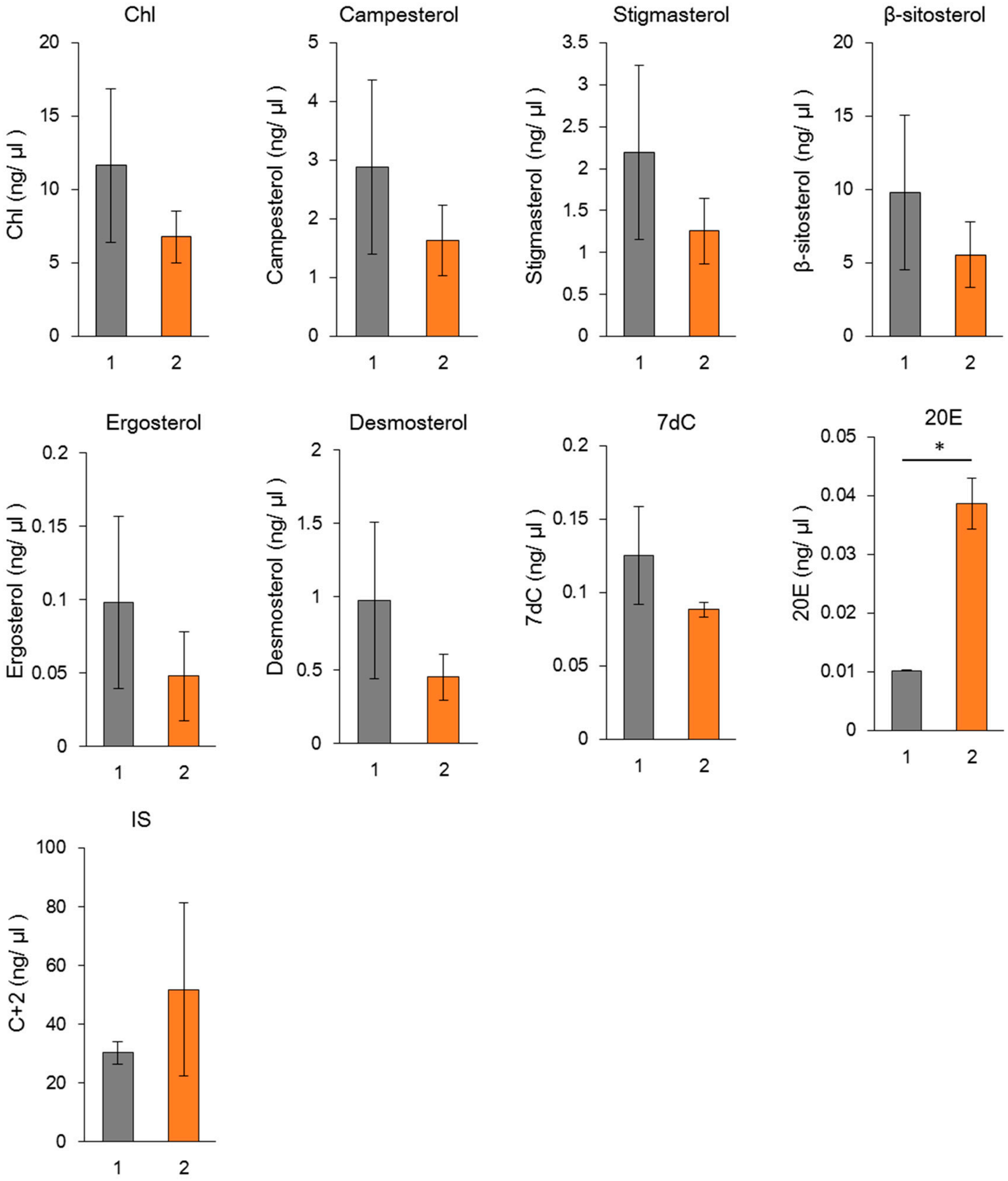

Figure S2. Comparison of two methods for sterol extraction from the hemolymph of the silkworm. Values are mean ± s. d. (n = 3). The sterols were extracted from the hemolymph of fifth instar larvae (Day 7) using different organic solvents as follows; [1] chloroform/methanol/hemolymph (10/10/9, v/v), [2] hemolymph/ methanol (1/9). Each sterol was quantified using LC-MS/MS as described in Materials and Methods section. Ecdysone was not detected from any samples. The statistical significant difference were determined by Student's-t test. *$p < 0.05$.

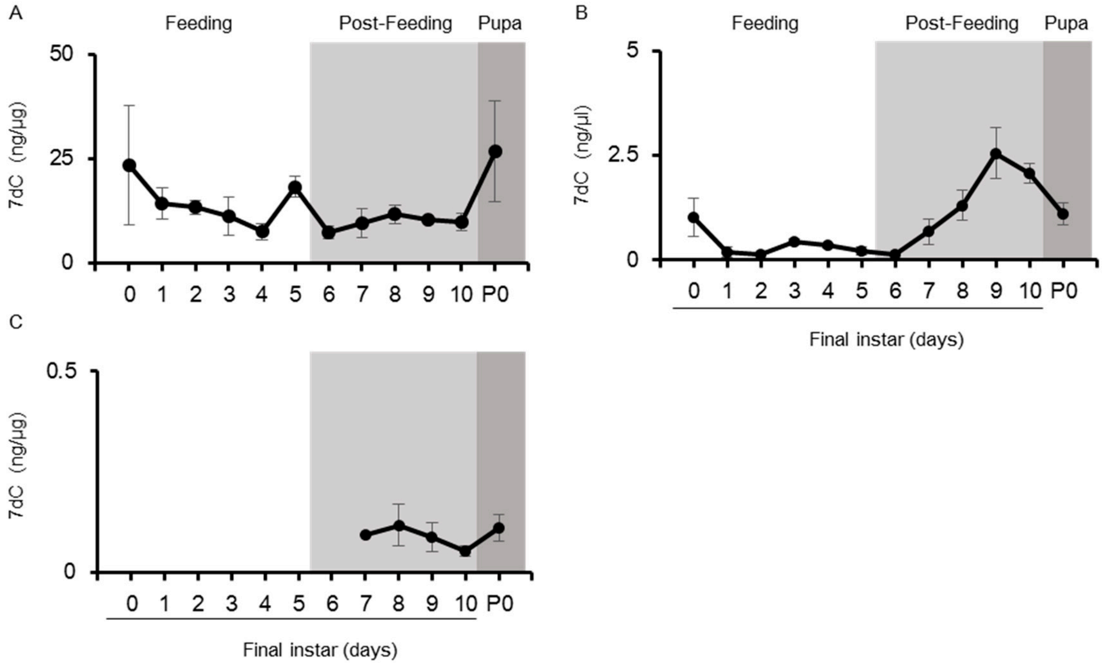

Figure S3. Change of the 7-dehydrocholesterol (7dC) amounts in silkworm tissues and the hemolymph. The amounts of 7dC in the PGs (**A**), the hemolymph (**B**), and the midgut (**C**). The 7dC amount in the PGs and midgut were normalized using the protein amounts of respective samples. Values are mean ± s.d. (n = 3).

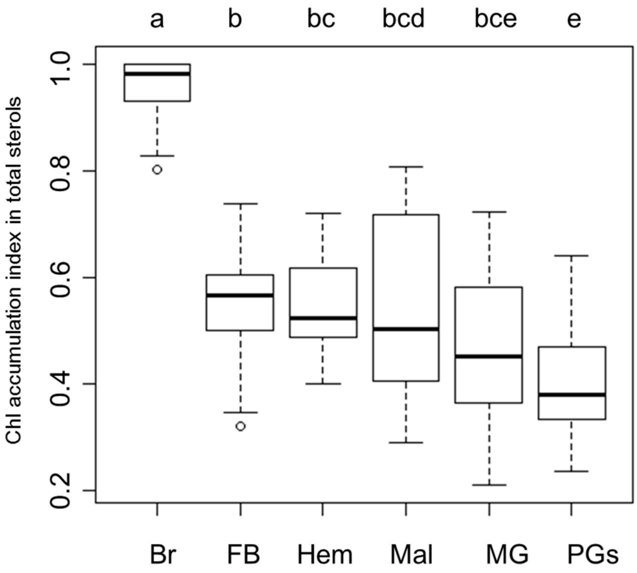

Figure S4. Comparison of Chl proportion to total sterols in silkworm tissues and the hemolymph. Chl accumulation index of total sterols showed Chl proportion against the total amount of detected sterols. The data in this figure are the same set as in Figure 2, 4, 5 and Supplementary Figure S3, S5-S7. The statistical significance of differences was determined by one-way analysis of variance (ANOVA)

followed by Tukey-Kramer's test. Br: brain, PGs: prothoracic glands, MG: midgut, FB: fat body, Mal: Malpighian tubules, Hem: hemolymph.

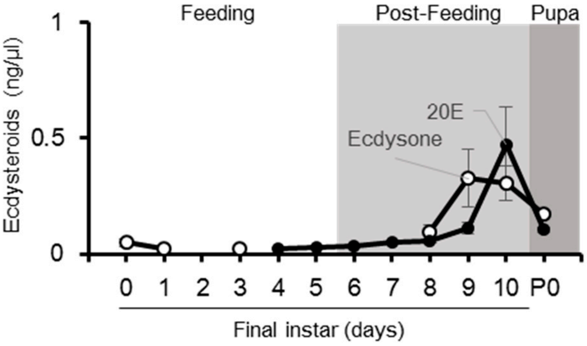

Figure S5. Changes of ecdysone and 20-hydroxyecdysone (20E) concentrations in the hemolymph. Ecdysone and 20E concentrations were quantified using LC-MS/MS as described in Materials and Methods section. Values are mean ± s.d. (n = 3). Blanks indicate "not detected".

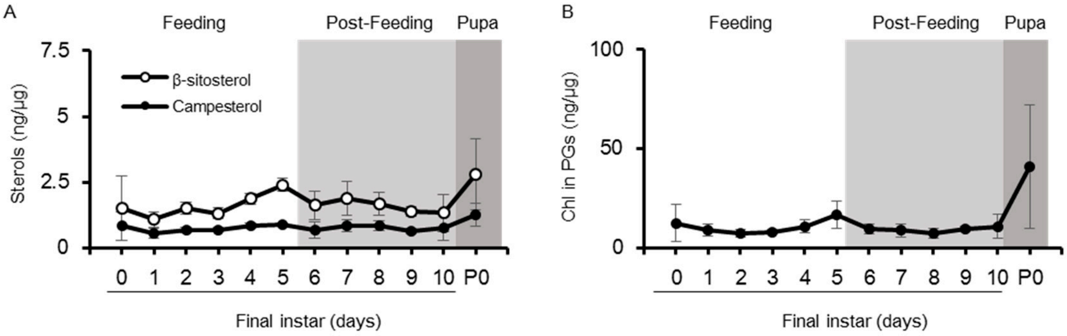

Figure S6. Changes of sterol amounts in the PGs from final instar to pupal periods. The amounts of dietary sterols (β-sitosterol and campesterols) (**A**) and Chl (**B**). Sterols were extracted from individual PGs and the amounts of sterols quantified using LC-MS/MS were standardized with the protein contents of respective samples. Stigmasterol, ergosterol, and desmosterol were not detected from the PGs. The amounts of 7dC in the PGs were shown in Supplemental Fig. S3. Values are mean ± s.d. (n = 3). P0: pupal stage day 0.

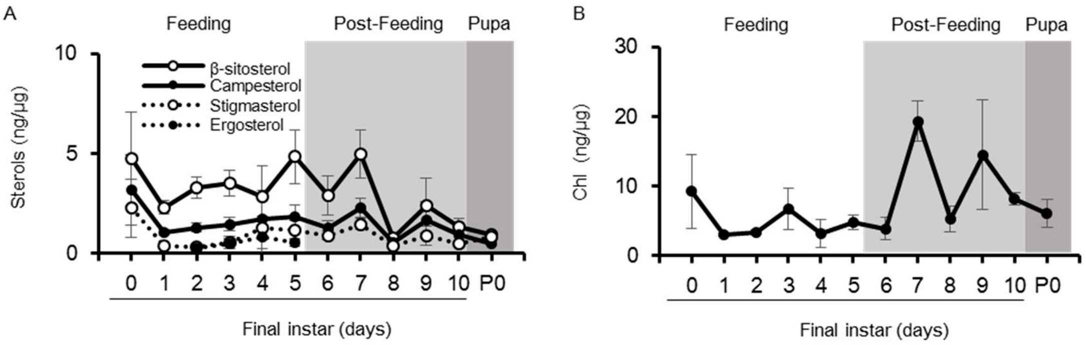

Figure S7. Changes of sterol amounts in Malpighian tubules from final instar to pupal periods. The amount of dietary sterols (A) and Chl (B). Sterols were extracted from Malpighian tubules and the amounts of sterols

quantified using LC-MS/MS were standardized with the protein contents of respective samples. Desmosterol and 7dC were not detected from Malpighian tubules. Values are mean ± s.d. (n = 3). P0: pupal stage day 0.

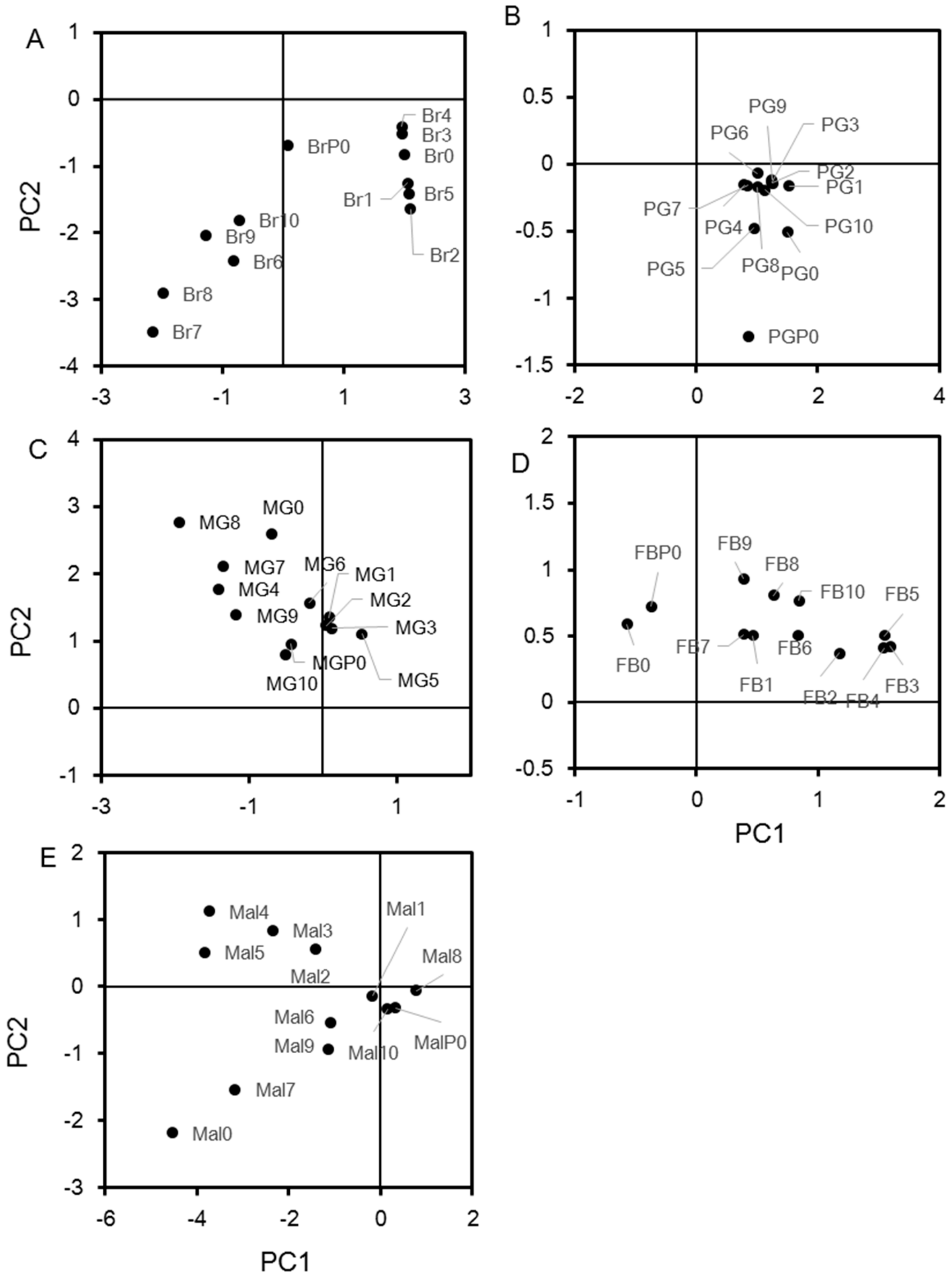

Figure S8. Characterization of insect tissues by sterol profiles with labels of developmental stages. The data were same set for Figure 7A, but labelled with developmental stages. Plots of each tissue were separated to

the brain (**A**), PGs (**B**), midgut (**C**), fat bodies (**D**), and Malpighian tubules (**E**). The vertical axis represents the first principal component and the horizontal axis represents the second one.
